# Crossmodal Interactions Between Olfaction and Touch Affecting Well-Being and Perception of Cosmetic Creams

**DOI:** 10.3389/fpsyg.2021.703531

**Published:** 2021-08-13

**Authors:** Sandra Courrèges, Rim Aboulaasri, Anjali Bhatara, Marie-Héloïse Bardel

**Affiliations:** ^1^Beauty Research and Performance, Innovation Research and Development Department, CHANEL Fragrance and Beauty, Pantin, France; ^2^IT&M Stats, Paris, France; ^3^Morard Editing, Chaville, France

**Keywords:** crossmodal, olfaction, tactile perception, well-being, cosmetics, liking, JARs

## Abstract

In the present series of studies, we investigated crossmodal perception of odor and texture. In four studies, participants tried two textures of face creams, one high viscosity (HV) and one low viscosity (LV), each with one of three levels of added odor (standard level, half of standard, or base [no added odor]), and then reported their levels of well-being. They also reported their perceptions of the face creams, including liking (global liking of the product, liking of its texture) and “objective” evaluations on just about right (JAR) scales (texture and visual appearance evaluations). In Study 1, women in France tried the creams on their hands, as they would when testing them in a store, and in Study 2, a second group of French women tried the creams on their faces, as they would at home. In Studies 3 and 4, these same two procedures were repeated in China. Results showed that both odor and texture had effects on well-being, liking, and JAR ratings, including interaction effects. Though effects varied by country and context (hand or face), the addition of odor to the creams generally increased reports of well-being, global liking and texture liking, in some cases affecting the “objective” evaluations of texture. This is one of the first investigations of crossmodal olfactory and tactile perception's impacts on well-being, and it reinforces previous literature showing the importance of olfaction on well-being.

## Introduction

Odor and texture are important factors for both the enjoyment and the pleasure gained from cosmetics. The present study focused on the interactions between odor and texture and their effect(s) on well-being resulting from application of face creams as well as liking and perception of the creams' textures. This experience was examined in two contexts, one like in a “store,” in which women tested a product by applying a face cream to their hands, and the other like at “home,” in which women applied cream to their faces. To explore cultural differences in this experience, similar versions of this study were performed in both France and China.

We usually think of each of our five senses as functioning separately; we hear a sound, smell an odor, or see colors. But more and more studies have found that interactions occur among these senses, influencing these seemingly basic perceptions. Vision can override other senses, causing viewers to believe they hear a voice coming from the mouths of actors on a cinema screen or a ventriloquist's dummy when the voice's real source is elsewhere (see Alais and Burr, 2004, for a discussion of bimodal integration). Taste, smell, texture, appearance, and even sounds can be important in the experience of a fine meal (see Spence, 2010 for a discussion of crossmodal influences on the experience of bacon-and-egg ice cream). Crossmodal associations are strong and pervasive; even when instructed to ignore stimuli from another sense, studies have demonstrated this other sense's influence, for example, of visual stimuli on olfactory perception (Demattè et al., 2009).

Generally, in the study of crossmodal associations, certain senses have been studied more than others. Many studies have examined multisensory perception through vision and olfaction (e.g., Barkat et al., 2003; Österbauer et al., 2005; Demattè et al., 2009; Seo et al., 2010; Zellner, 2013; Guerdoux et al., 2014; Robinson et al., 2015; Nehmé et al., 2016), vision and tactile perception (Duncan et al., 2020), and audition and olfaction (e.g., Belkin et al., 1997; Seo and Hummel, 2011; Crisinel and Spence, 2012). Multisensory integrations between texture and olfaction have been studied together extensively in gustatory/olfactive perception and food texture (e.g., Hollowood et al., 2002; Saint-Eve et al., 2006; Bult et al., 2007; Roudnitzky et al., 2011).

To our knowledge, there have only been three other studies to have examined the multisensory interaction of tactile perception and olfaction outside the domain of food/flavor perception, and these along with a fourth study (the one on vision and tactile perception mentioned above) are especially relevant to the present report. In the first, the authors demonstrated a clear influence of odor on perception of texture: they tested participants' perception of the softness of fabric treated with different chemicals in two separate odor contexts: lemon vs. animal scent (Exp. 1, odor presentation carefully controlled) and lavender vs. animal scent (Exp. 2, odor added directly to fabric) and in both cases the vegetal scent resulted in higher softness ratings of the fabric (Demattè et al., 2006).

The second article to examine odor and tactile perception focused on the influence of taste. One odor was sampled in a viscous solution, a second in a sweet-viscous solution, and a third in water. When later sniffed alone, the odor paired with the sweet-viscous solution was judged as sweeter and thicker than the others, but the odor paired with the viscous solution was not rated significantly differently from the others, though there was a trend toward this (Stevenson and Mahmut, 2011). The authors hypothesized that the experience with the sweet taste may have enhanced learning of the odor-viscosity pairing. The third article to examine olfaction and tactile perception found that a pleasant touch was rated as less pleasant when participants were exposed to a disgusting odor (feces), though a pleasant odor (rose) had no effect on ratings (Croy et al., 2014).

The final study, like the present experiments, focused on perception of viscosity of a skin care product (Duncan et al., 2020), demonstrating clear neurological and behavioral effects of vision on perception of texture. Participants showed brain activation in somatosensory regions in response to visual-only texture cues (a video of the lotion being poured into a petri dish), suggesting early crossmodal perception of texture. Behaviorally, the simultaneous presentation of a more viscous lotion visual cue with lotion application made participants judge the feeling of both the viscous and watery lotion as more moisturizing.

Though tactile perception and olfaction have not often been examined together outside the context of food, olfaction as a domain is particularly rich in crossmodal correspondences, that is, tendencies for an odor to be associated with a feature in a distinct sensory modality. Odor-color, odor-taste, and probably some odor-texture mappings can be explained by associative learning, but others such as common odor-sound associations are more complex (Deroy et al., 2013).

### Why Study Tactile Perception and Odor Specifically?

It is interesting to study tactile perception and odor together for two reasons: First, given the prevalence of scented cosmetic products with varying textures, olfaction and tactile perception together offer an ecologically-valid way to examine the impacts of one sensory input on another and multisensory perception on emotion and well-being. Second, both of these senses develop early in gestation, with some markers of system maturity present around the 29th week (Hrbek et al., 1973; Chuah and Zheng, 1987), and, perhaps most importantly, they are *emotionally* important from the beginning. Tactile stimulation and massage can be used to reduce pain and anxiety and increase weight gain in preterm neonates (e.g., Field et al., 1986). And, though the valence of emotional responses to odors appears to be learned (see Herz, 2002 for a review) the olfactory system is fully developed at birth (Chuah et al., 2003) and clear emotional responses to odors are present from this time (Steiner, 1974). The primacy of these senses may be a reason that they are both so closely linked to pleasure and comfort, and through pleasure to well-being, even in adulthood.

According to Aristotle, well-being could be thought of as a combination of at least two components: hedonia (pleasure) and eudaimonia, or finding meaning in life (Berridge and Kringelbach, 2011; Dolcos et al., 2018). Odors act mainly on the former—an odor's experience is difficult to dissociate from its hedonic tone (Schiffman et al., 1995; Rétiveau et al., 2004)—but they are also tightly linked to autobiographical memories (for a review, see Chu and Downes, 2000) and can be even more effective than visual cues for triggering autobiographical memories (de Bruijn and Bender, 2018). A classic example is Proust's madeleine. One might imagine that the recall of autobiographical memories could have an impact on eudaimonia; indeed, a recent review suggests that odor-invoked autobiographical memories can increase positive emotions and reduce negative emotions and stress (Herz, 2016). Even so, odors' main role in well-being is likely to be on the hedonic side.

Multiple examples exist of odors' hedonic influences on well-being. Aromatherapy, a healing technique involving inhaling or using essential oils on the skin, may provide a promising avenue of research for treating psychiatric disorders (Perry and Perry, 2006) and odors themselves have effects on mood, physiology, and behavior (Marchand and Arsenault, 2002; Herz, 2009). Pleasant odors can be used in classical conditioning to positively influence human behavior (Chu, 2008). Unpleasant odors, while not increasing pain intensity, can make the experience of pain more unpleasant (Villemure et al., 2003) and, similarly, sweet odors (note that this result does not extend to all pleasant odors) do not decrease pain intensity but can increase pain tolerance (Prescott and Wilkie, 2007). Marchand and Arsenault (2002) did find a reduction in pain perception, and this effect was gender-specific; pleasant odors reduced pain perception for women but not men, though mood was improved in both groups.

The addition of pleasant odors to cosmetic products has been shown to increase participants' ratings of both pleasantness and arousal (Barkat et al., 2003), though pleasantness ratings can be affected by external factors such as an impression of luxury associated with the odorant (Baer et al., 2018) or the odorant's name (Porcherot et al., 2012). In general, measuring the effect of odors on mood or affect is a challenge; several studies of odor and consumer behavior have reported no positive effects (Ellen and Bone, 1998) or no difference at all between scented and unscented conditions (Morrin and Ratneshwar, 2000, 2003) on self-reported affect. This may be due to changes in mood due to scent being below participants' awareness level (see Rimkute et al., 2016 for a discussion). This implies that direct self-report may not be the best way to evaluate changes in affect or mood in more real-life situations. An alternative explanation is that this lack of effects is due to emotion measurement instruments not being well-adapted to emotions from odors. Researchers in Geneva have investigated the best means to evaluate these emotions, creating Emotion and Odor Scales based on behavioral studies in different countries (Chrea et al., 2009; Ferdenzi et al., 2011) as well as a universal scale combining data from all of these (Ferdenzi et al., 2013a).

The present study attempts to build on previous research in order to investigate the interaction of odors and textures in an ecologically valid, “as in real life” context. When consumers want to choose a new skincare product, they often go to specialized beauty stores or department stores and ask to test the different products that are available. Even if they are products meant to be applied to the face, in general, consumers test them on their hands. We attempted to replicate this experience, though in a controlled environment. We report results from women who tested creams with two different textures (low and high viscosity) and three levels of odor (no added scent, half of the standard scent, and the standard scent level). One group tested these on their hands, as in a store, and another on their faces, as they would after having purchased the product, at home. The participants rated both their liking of the creams and how it made them feel, as well as giving more objective measures such as JARs (Just About Right) of the texture and appearance. Rather than a single identifiable odor such as lavender, anise, or lemon, we used a floral blend comparable to others found in commercially available cosmetics. The proportions of odor tested were also based on those commercially available.

This study is designed to answer questions about the effects of odor and texture on well-being, liking, and JAR ratings of face cream. (1) Does the percentage of added odor affect well-being ratings for a cream after applying it to the skin? Does its effect vary with texture? (2) Does the percentage of added odor affect perception of the product, in terms of liking and “objective” evaluation? How does its effect vary with texture? (3) Do these effects differ depending on whether the cream is applied to the hand or the face? Finally, (4) do we find similar effects across different cultures?

## Study 1: Hand Application In A French Sample

### Materials and Methods

#### Participants

A group of 60 French-speaking women completed this task (*M*_age_ = 45.7, age range = 31–60). All participants lived in the Paris region. None were pregnant, and none had participated in other cosmetic tests in the previous three months. They all used skincare products on a daily basis, purchased at specialized beauty stores, and they were asked what brand of skincare they use at the beginning of the procedure to verify that they used higher-end or luxury brands. They were recruited by and tested at Eurosyn, a sensory testing laboratory which recruits from a list of participants in the community. The participants were not aware that it was an experiment run by Chanel. Participants were reimbursed for their time with gift cards. All research was conducted according to the principles expressed in the Declaration of Helsinki, and written informed consent was obtained from every participant. All the data collected respond to GDPR requirements (European Chart for General Data Protection Regulation) and all the products tested by the participants were validated by a toxicologist (with a Safety Certificate for each product).

#### Materials

##### Creams

**Figure 1:**
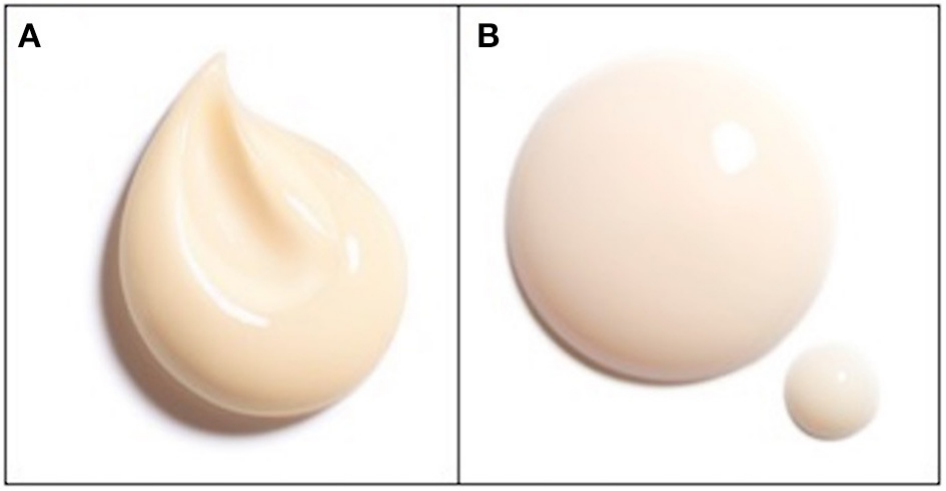
Photographs of two creams showing texture difference. **(A)** high-viscosity, **(B)** low-viscosity.

The materials used in this study were face creams with two different textures (low and high viscosity), each with three levels of odor, 0% (Base), 0.15% (Half of standard added odor), or 0.3% (Standard added odor). Viscosity of the creams was measured as its elastic modulus using oscillatory rheometry. The high-viscosity (HV) cream had a *G'* of 633 Pa, the low-viscosity (LV) cream had a *G'* of 220 Pa. These two types of viscosity are clearly distinguishable both visually (see Figure 1) and by touch for naïve participants. Viscosity did not vary with added odor. The type of odor added to the creams (a proprietary blend with a floral scent) did not vary, only its concentration. However, the Base condition was not neutral; each ingredient of the cream had its own odor, and one of the active components of the cream had a vanilla scent. The odors of these raw materials were modulated by the additional odor in the Half and Standard conditions. These levels of odor were chosen in order to compare the actual scent (Standard) products to the same products without added scent (raw odor), to see if the addition of a scent is beneficial for the perception of the product and the emotions felt with it. The third product version (Half) was chosen in order to compare the Standard products to a product containing less added perfume but in which the raw odor was nonetheless disguised. This allowed us to examine whether a different concentration of perfume would impact the perception of the product and the emotions felt with it.

##### Questionnaire

The questionnaire (completed only after testing the cream) was presented on a computer using the program Fizz. Participants responded on Visual Analogue Scales by clicking at any point along a line between two extremes, which varied by question. Responses were recorded by the software as values between 0 and 10. The questions discussed here were originally in French but have been translated to English for this report. Questions concerned their liking of the product, Just About Right measures (JARs), and their emotional state (based on the Emotion and Odor Scales [EOS]; Chrea et al., 2009). The questions analyzed for the present report were (1) How much did you like the product? (Not at all—Very much), (2) How much did you like the texture of the cream? (Not at all—Very much), (3) How would you characterize the texture of the cream? (Too light—[Just right]—Too oily), (4) How would you characterize the appearance of the cream? (Too transparent—[Just right]—Too opaque), (5) How strongly do you feel each emotion? (Not at all—Extremely; “well-being” was the emotion analyzed for the present report).

#### Procedure

As the study aimed to investigate emotional states linked to products, participant fatigue was likely if all 6 products were tested in one session. Therefore, the study occurred over two sessions of 45 mins each, and there was a minimum of 48 h between sessions. In each session, participants tested one of the textures at all three levels of odor concentration. The order of presentation within a session was counterbalanced across participants.

The day of the study, participants were asked to abstain from applying any scented body products or perfume. In each session, participants were told “You will be testing three premium face creams on your hand, as you would when testing it in a store before purchasing it. After each of the three tests, you will respond to several questions concerning the cream you've just tried.” The creams were delivered to the backs of participants' hands by an experimenter using pre-filled syringes containing 0.1 ml. This procedure is like what happens in stores, though vendors use a spatula instead of a syringe. All the creams were delivered without any information concerning the product (scent percentage, brand, ingredients, skin benefits), except that it was a “premium facial cream.”

After testing each cream, participants filled out the questionnaire, including the questions described above. The instructions for the questionnaire reminded them to keep in mind that the cream was intended for use on the face, even though they had tested it on their hands. They then took a break for 10 mins before trying the subsequent cream; during this time, they were asked to thoroughly wash their hands using unscented soap.

**Figure 2:**
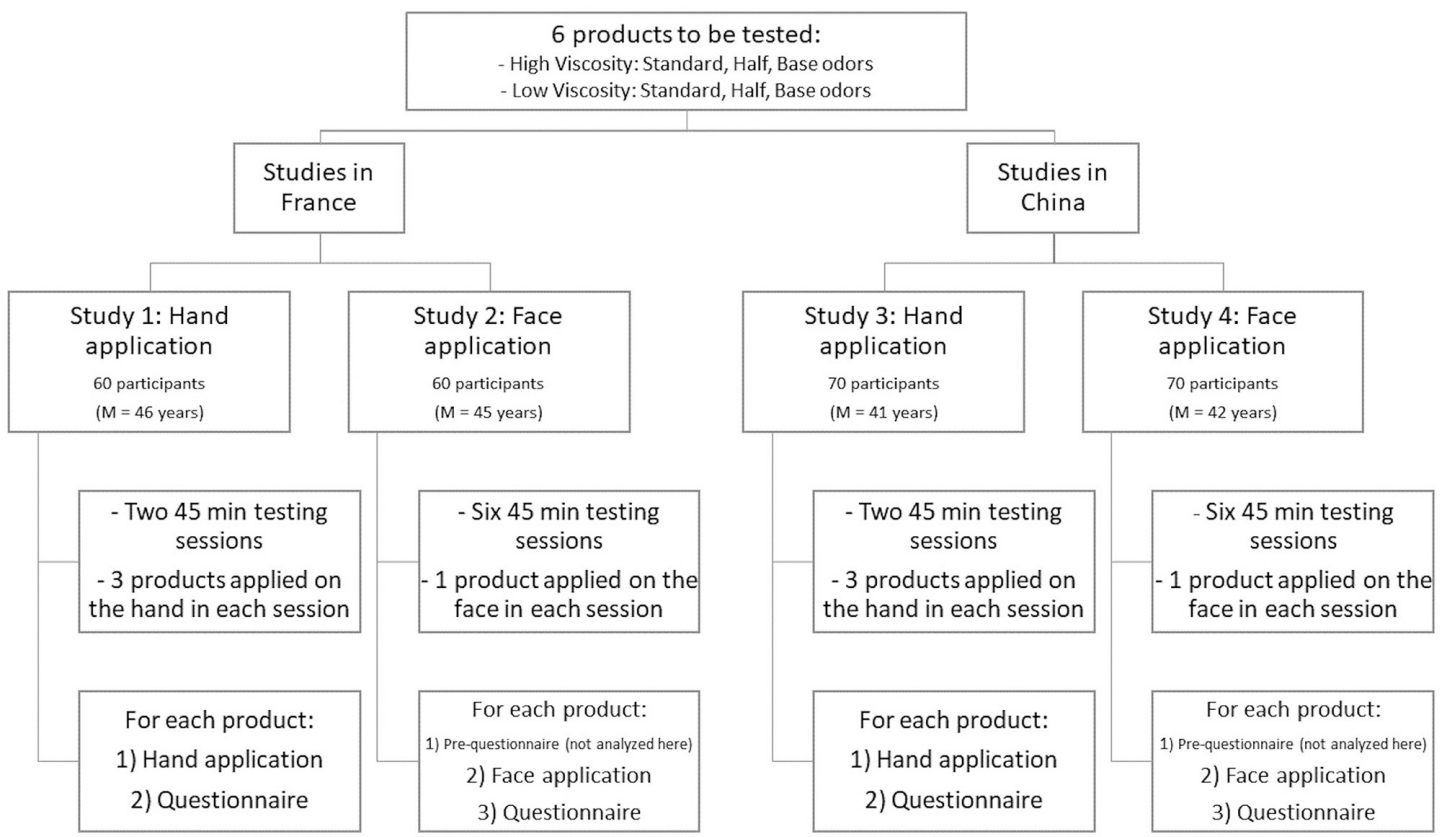
Diagram of the methods for all four studies.

See Figure 2 for an outline of the experimental procedures for all four studies.

#### Analyses

Overall analyses were the same for all four studies. The dependent variables of interest were examined using ANOVAs (type III) to compare nested generalized linear mixed models fitted using the statistics program R, version 4.0.3 (R Core Team, 2020), and the package lme4, version 1.1.26 (Bates et al., 2015, R package: lme4, RRID:SCR_015654). Fixed effects variables were texture and odor, and subject was included as a random factor. Additional factors were tested in preliminary models (age, reported skin type), and none of these resulted in better models so they were not explored further. Participants' continuous ratings were quantified on a scale of 0–10. Because the different textures may have changed the odor of the creams, we do not report results on perception of odor, as these would not be possible to interpret. We verified that the addition of odor did not change the viscosity of the cream. Because the factor odor had three levels, Standard and Half odor were each compared to the Base odor baseline within the model, and the comparison between Standard and Half odor was done using a pairwise *post-hoc* test using the package lmerTest, version 3.1.3 (Kuznetsova et al., 2017, R package: lmerTest, RRID:SCR_015656) in R. All three of these comparisons are reported including a Bonferroni correction of their *p*-values.

### Results

Descriptive statistics (means, SD, ranges) for all the ratings are shown in Table 1. Ratings of well-being after trying the product were affected by odor, χ^2^(2) = 45.6, *p* < 0.001. The Bonferroni-corrected model showed differences between Standard and Base, β = 2.38, *SE* = 0.38, *t*_(299)_ = 6.25, *p* < 0.001, and between Half and Base, β = 2.05, *SE* = 0.38, *t*_(299)_ = 5.38, *p* < 0.001, but not between Standard and Half, β = 0.27, *SE* = 0.27, *t*_(299)_ = −0.99, *p* = 0.64. There was no main effect of texture, χ^2^(1) = 1.38, *p* = 0.24, nor was there a significant interaction, χ^2^(2) = 0.71, *p* = 0.70.

**Table 1:** Descriptive statistics for each level of viscosity and odor in all four studies.

			**Liking**				**Just about right ratings**				
		**Well-being**		**Product**		**Texture**		**Oiliness**		**Opacity**	
		**Mean (SD)**	**Range**	**Mean (SD)**	**Range**	**Mean (SD)**	**Range**	**Mean (SD)**	**Range**	**Mean (SD)**	**Range**
Study 1: France/Hand application	LV cream	5.62 (3.00)	1–10	6.89 (2.51)	0–10	7.01 (2.43)	0–10	4.64 (1.13)	0–8.7	4.80 (0.80)	0–7.1
	HV cream	5.81 (2.80)	0–10	6.97 (2.38)	0.2–10	6.93 (2.42)	0.4–10	6.02 (1.63)	0.7–10	5.14 (0.88)	1–9.9
	Base odor	4.36 (3.07)	0–9.9	5.67 (2.74)	0–10	6.29 (2.67)	0–10	5.21 (1.93)	0–10	4.77 (1.16)	0–9.7
	Half added odor	6.25 (2.61)	0–10	7.34 (2.09)	0.7–10	7.13 (2.28)	0.4–10	5.36 (1.44)	0.3–9.5	5.07 (0.64)	2.5–8.8
	Std added odor	6.52 (2.51)	0–10	7.78 (1.90)	0.6–10	7.49 (2.14)	0.4–10	5.42 (1.26)	2.7–9.8	5.06 (0.64)	2.7–9.9
Study 2: France/Face application	LV cream	7.23 (1.75)	1–10	7.13 (1.87)	1.1–10	7.37 (2.02)	1.1–10	4.69 (1.26)	0.7–8	4.85 (0.85)	1–7.6
	HV cream	6.36 (2.33)	0–10	5.72 (2.69)	0–10	5.22 (3.08)	0–10	7.38 (1.75)	3–10	5.44 (0.98)	0.6–8.6
	Base odor	6.46 (2.12)	1–10	5.84 (2.43)	0.1–10	5.88 (2.89)	0–10	6.04 (2.18)	0.8–10	5.08 (1.15)	0.6–8.6
	Half added odor	6.82 (2.10)	0.8–10	6.57 (2.36)	1–10	6.24 (2.80)	0–10	6.19 (2.00)	0.7–10	5.20 (0.85)	1.1–8.1
	Std added odor	7.12 (2.05)	0–10	6.87 (2.37)	0–10	6.77 (2.70)	0–10	5.89 (1.91)	1–10	5.15 (0.87)	1.6–8.3
Study 3: China/Hand Application	LV cream	5.27 (2.88)	0–10	6.75 (2.15)	0.8–10	7.33 (1.89)	1.5–10	4.76 (1.04)	0.6–8.2	4.90 (0.87)	1.1–8.2
	HV cream	5.69 (2.78)	0–10	7.53 (1.81)	0.8–10	7.55 (1.87)	0.5–10	5.86 (1.21)	3.8–10	5.35 (0.93)	0.3–9.2
	Base odor	5.19 (2.78)	0–10	6.62 (2.18)	0.8–10	7.13 (1.99)	0.5–10	5.17 (1.15)	1.7–9	5.12 (1.01)	1.1–9.2
	Half added odor	5.62 (2.77)	0–10	7.14 (2.06)	0.8–10	7.43 (1.95)	0.9–10	5.18 (1.07)	1.7–8.8	5.04 (0.86)	0.3–8.6
	Std added odor	5.64 (2.95)	0–10	7.65 (1.69)	3–10	7.76 (1.66)	2–10	5.56 (1.47)	0.6–10	5.21 (0.92)	2.1–9.2
Study 4: China/Face application	LV cream	7.75 (1.42)	3.2–10	7.67 (1.54)	1.9–10	7.86 (1.48)	1.7–10	5.15 (0.73)	2.8–8.7	5.15 (0.70)	1.4–8.3
	HV cream	7.75 (1.51)	2.3–10	7.56 (1.53)	2.6–10	7.47 (1.74)	1.7–10	5.98 (1.19)	3.4–9.2	5.42 (0.93)	1.7–9.5
	Base odor	7.65 (1.58)	2.4–10	7.43 (1.69)	2.2–10	7.54 (1.69)	1.7–10	5.55 (1.16)	2.8–9.2	5.27 (0.88)	1.7–9.4
	Half added odor	7.89 (1.32)	3.4–10	7.70 (1.45)	1.9–10	7.69 (1.64)	1.7–10	5.54 (1.02)	3.4–9.1	5.38 (0.80)	3.7–8.7
	Std added odor	7.71 (1.48)	2.3–10	7.71 (1.44)	2.6–9.7	7.76 (1.56)	2.2–10	5.61 (1.03)	3.7–9.2	5.21 (0.82)	1.4–9.5

Overall liking of the cream was significantly affected by texture, β = 5.27, *SE* = 0.29, χ^2^(1) = 5.44, *p* = 0.02, with a preference for the HV cream, and odor, χ^2^(2) = 67.0, *p* < 0.001. Using a Bonferroni correction, the model showed significant differences between Standard and Base, β = 2.55, *SE* = 0.34, *t*_(300)_ = 7.42, *p* < 0.001, and between Half and Base, β = 2.3, *SE* = 0.34, *t*_(300)_ = 6.70, *p* < 0.001, but there was no difference between Standard and Half odor, β = 0.44, *SE* = 0.24, *t*_(300)_ = −1.81, *p* = 0.14. There was also an interaction between texture and odor, with Half and Standard differing from Base, β_Half_ = 0.39, *SE*_Half_ = 0.49, β_Standard_ = −0.88, *SE*_Standard_ = 0.49, χ^2^(2) = 7.14, *p* = 0.03.

**Figure 3:**
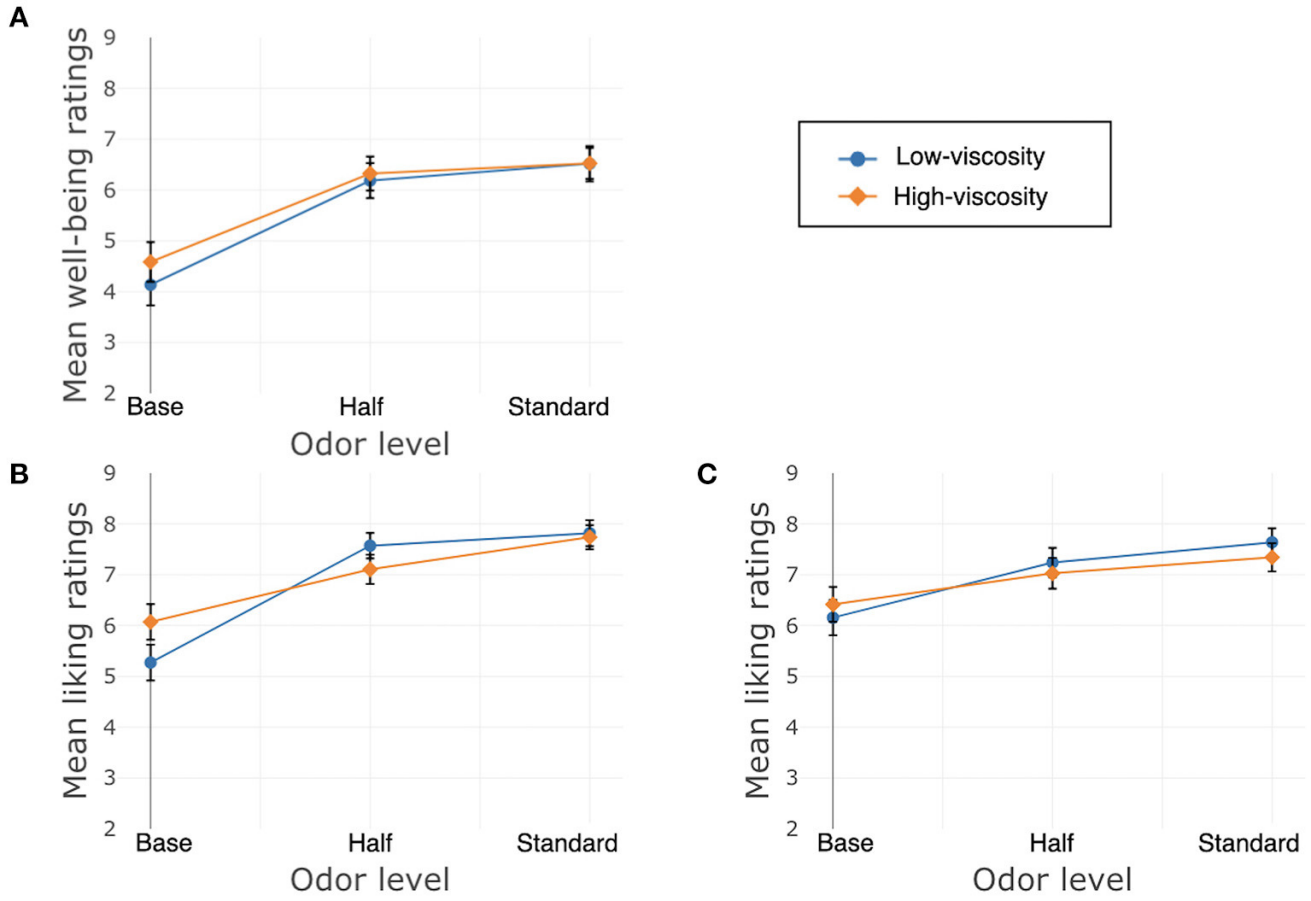
Study 1, France/Hand application, **(A)** well-being, **(B)** liking of the product, **(C)** liking of the texture. Error bars indicate standard error.

Liking of the texture of the cream was significantly affected by its odor, χ^2^(2) = 17.22, *p* < 0.001. The Bonferroni-corrected model showed differences between Standard and Base, β = 1.48, *SE* = 0.37, *t*_(300)_ = 4.01, *p* < 0.001, and between Half and Base, β = 1.08, *SE* = 0.37, *t*_(300)_ = 2.93, *p* = 0.008, but not between Standard and Half, β = 0.36, *SE* = 0.26, *t*_(300)_ = −1.37, *p* = 0.34. There was no main effect of texture, χ^2^(1) = 0.49, *p* = 0.48, nor was there a significant interaction between texture and odor, χ^2^(2) = 1.31, *p* = 0.52. See Figure 3 for well-being and liking ratings.

**Figure 4:**
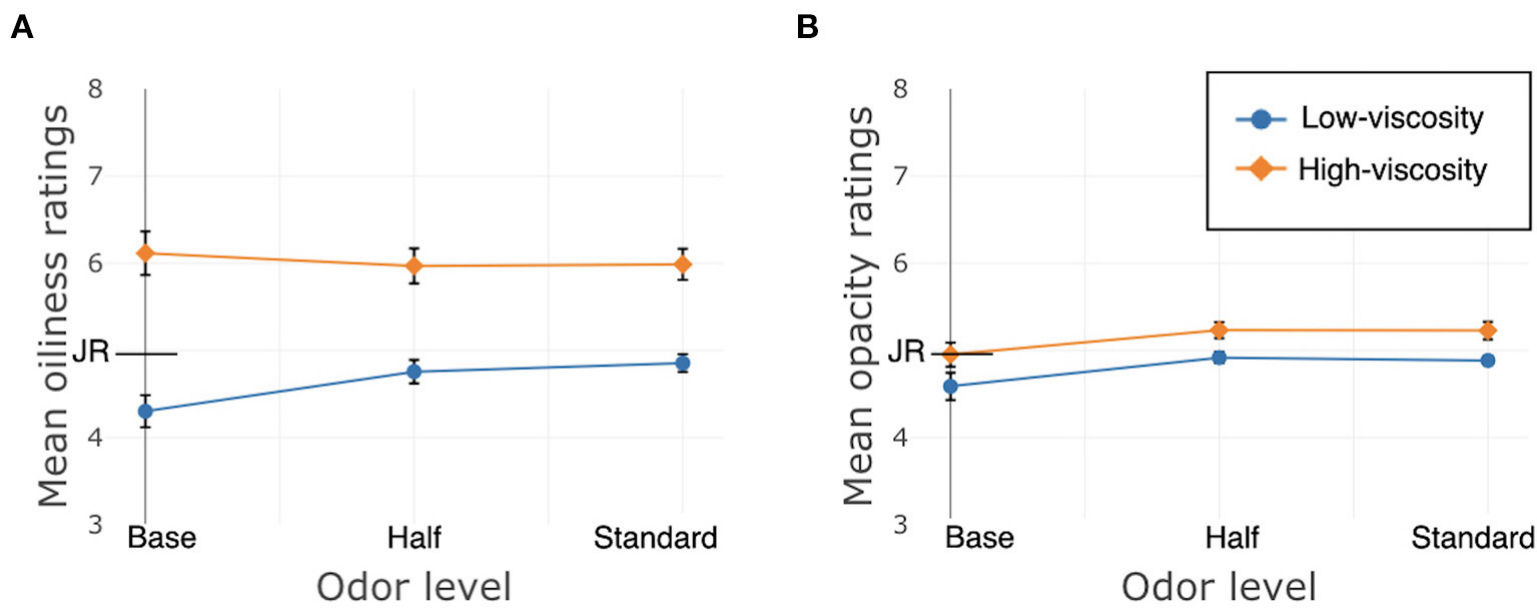
Study 1, France/Hand application, JAR ratings. JR, just right. **(A)** Oiliness, **(B)** Opacity. Error bars indicate standard error.

Just About Right (JAR) ratings of oiliness and opacity were affected by texture, Oiliness: β = 1.82, *SE* = 0.23, χ^2^(1) = 64.32, *p* < 0.001; Opacity: β = 0.37, *SE* = 0.13, χ^2^(1) = 7.88, *p* = 0.005, as well as odor, Oiliness: χ^2^(2) = 6.81, *p* = 0.03; Opacity: χ^2^(2) = 3.04, *p* = 0.02. See Table 2 for the model estimates for these two JAR ratings for odor. For Oiliness, there was a significant difference between Standard and Base, but Half did not differ from either, and for Opacity, Standard and Half differed from Base not from each other. The interaction between the factors was not significant for Opacity, χ^2^(2) = 0.07, *p* = 0.96, and it approached significance for Oiliness, β_Standard_ = −0.69, *SE*_Standard_ = 0.32, χ^2^(2) = 5.42, *p* = 0.07. See Figure 4.

**Table 2:** Study 1, France/Hand application: model estimates of the different levels of odor for each JAR rating.

		**β**	**Std. Err**.	***df***	***t***	***p***	**Significance**
Oiliness	Standard–Base	0.55	0.23	300	2.45	0.03	^*^
	Half–Base	0.46	0.23	300	2.01	0.09	
	Standard–Half	0.06	0.16	300	−0.37	0.72	
Opacity	Standard–Base	0.30	0.13	300	2.28	0.04	^*^
	Half–Base	0.33	0.13	300	2.54	0.02	^*^
	Standard–Half	0.02	0.09	300	0.21	0.84	

* *p <0.05*.

### Discussion

Results show that participants gave generally higher well-being ratings to creams with added odor, and there was no difference between the two textures. Participants also gave higher ratings of product liking to creams with added odor relative to those without. It also eliminated differences between textures: for the Base condition, the HV cream was rated higher, but there was no difference between the two textures for Half and Standard odor. Interestingly, for liking of texture, there was no difference between the two levels of viscosity. Only odor had an effect, with higher liking for creams with added odor.

The texture and color JAR ratings (oiliness and opacity) were affected by both texture and odor, with higher ratings (i.e., oilier and more opaque) for the HV cream, as expected. What was unexpected was that the addition of odor also resulted in generally oilier and more opaque ratings. Though there were no significant interactions, the interaction between texture and odor for oiliness approached significance. The trajectory of the lines, both moving toward the center of the scale, suggests a tendency for both of the creams' ratings to approach “Just About Right” with added odor.

In Study 2, participants tested these same creams on their faces to evaluate whether this would result in differences in ratings.

## Study 2: Face Application In A French Sample

### Materials and Methods

#### Participants

A group of 60 French-speaking women completed this task (*M*_age_ = 44.6, age range = 30–60). The recruitment criteria and testing laboratory procedures were the same as in Study 1. A random selection of 32 participants were chosen to undergo a prosody-measurement task as well, before and after the experimental procedure. This task was performed in order to measure vocal variation while reading a neutral text, which can serve as an indicator of emotional feelings. These data are not analyzed for the present report.

#### Materials

##### Creams

The creams used in this study were the same as in Study 1

##### Questionnaires

Participants completed questionnaires developed specifically for this study before and after product application. As in Study 1, participants responded in the program Fizz by placing a cursor anywhere along a continuous line between two extremes, which varied by question. All questions analyzed in the present study were from the post questionnaire: well-being, liking and JAR ratings of the face creams, and were the same as in Study 1, with the exception of the well-being question. Given previous research showing that mood states in response to odors may sometimes be below the threshold of perception (Rimkute et al., 2016), we chose to ask more indirectly about well-being. The participants rated their agreement with the statement “With this cream I have the impression of doing myself some good.”

#### Procedure

The study aimed to investigate emotional states linked to products, so in order to prevent participant fatigue, we did not test the products in a single session. The study occurred over six sessions of 45 mins each, and there was a minimum of 48 h between sessions. For each of the six sessions, participants were presented with only one texture/odor combination. The order of presentation was counterbalanced across participants.

The day of the study, participants were asked to abstain from applying any scented body products or perfume. They either arrived without makeup or were asked to arrive early to remove their makeup. In the latter case, a few minutes were left after makeup removal to let their skin rest before the experimental procedure began. They were told, “You will apply a premium cream to your face as you would do at your home. Before and after application, you will respond to different questions.”

In each session, *N* = 28 participants began by completing the pre questionnaire. The *N* = 32 who had been randomly selected for the prosody task began with it (reading a short page of text aloud; these data are not presented in the current paper) and completed the pre questionnaire after. Before the questionnaire, they washed their hands with unscented soap. At the end of the questionnaire, all participants were asked to look at their skin and overall facial appearance in the mirror.

In Study 1, participants were given a specific amount of cream in a syringe, as if they were trying a sample in a store. In Study 2, to recreate a more home-like experience, the face creams were given to participants in sample sized containers, the HV cream in a round jar with a screw top lid and the LV cream in a jar with a pump. All the jars were “blind lab samples,” without any information concerning the product (such as scent percentage, brand, ingredients, skin benefits), except that it was a “premium facial cream.” Participants were asked to apply the cream the same way they would do it at home, and they were free to choose the amount they needed for their face.

The post questionnaire was completed again after product application. After this, either the session ended (for *N* = 28) or the participant completed the prosody task for the second time (*N* = 32). In the sixth and final session, participants completed the final questionnaire after either the post questionnaire or after the prosody task, whichever was the final task for the participant.

#### Results

Descriptive statistics (means, SD, ranges) for all the ratings are shown in Table 1. The rating of well-being, “does me some good,” was affected by both texture, β = −0.72, *SE* = 0.29, χ^2^(1) = 6.16, *p* = 0.01 and odor, χ^2^(2) = 6.29, *p* = 0.04, but the interaction was not significant, χ^2^(2) = 0.85, *p* = 0.65. Participants gave higher ratings after trying the LV cream. For the three levels of odor, there were only significant differences between the Standard and Base conditions, β = 0.70, *SE* = 0.29, *t*_(300)_ = 2.40, *p* = 0.04. Half did not differ significantly from either: for the comparison between Half and Base, β = 0.54, *SE* = 0.29, *t*_(300)_ = 1.84, *p* = 0.14, and for Standard versus Half, β = 0.31, *SE* = 0.21, *t*_(300)_ = 1.47, *p* = 0.28.

Overall liking of the face cream was significantly affected by its texture, β = −1.25, *SE* = 0.36, χ^2^(1) = 11.86, *p* = 0.001, with higher liking ratings for the LV cream, and odor, χ^2^(2) = 10.07, *p* = 0.007. Using a Bonferroni correction, the model showed significant differences between Standard and Base, β = 1.03, *SE* = 0.36, *t*_(300)_ = 2.82, *p* = 0.01, and between Half and Base, β = 0.97, *SE* = 0.36, *t*_(300)_ = 2.68, *p* = 0.02, but there was no difference between Standard and Half, β = 0.30, *SE* = 0.26, *t*_(300)_ = −1.17, *p* = 0.48. There was no interaction between texture and odor, χ^2^(2) = 1.24, *p* = 0.5.

**Figure 5:**
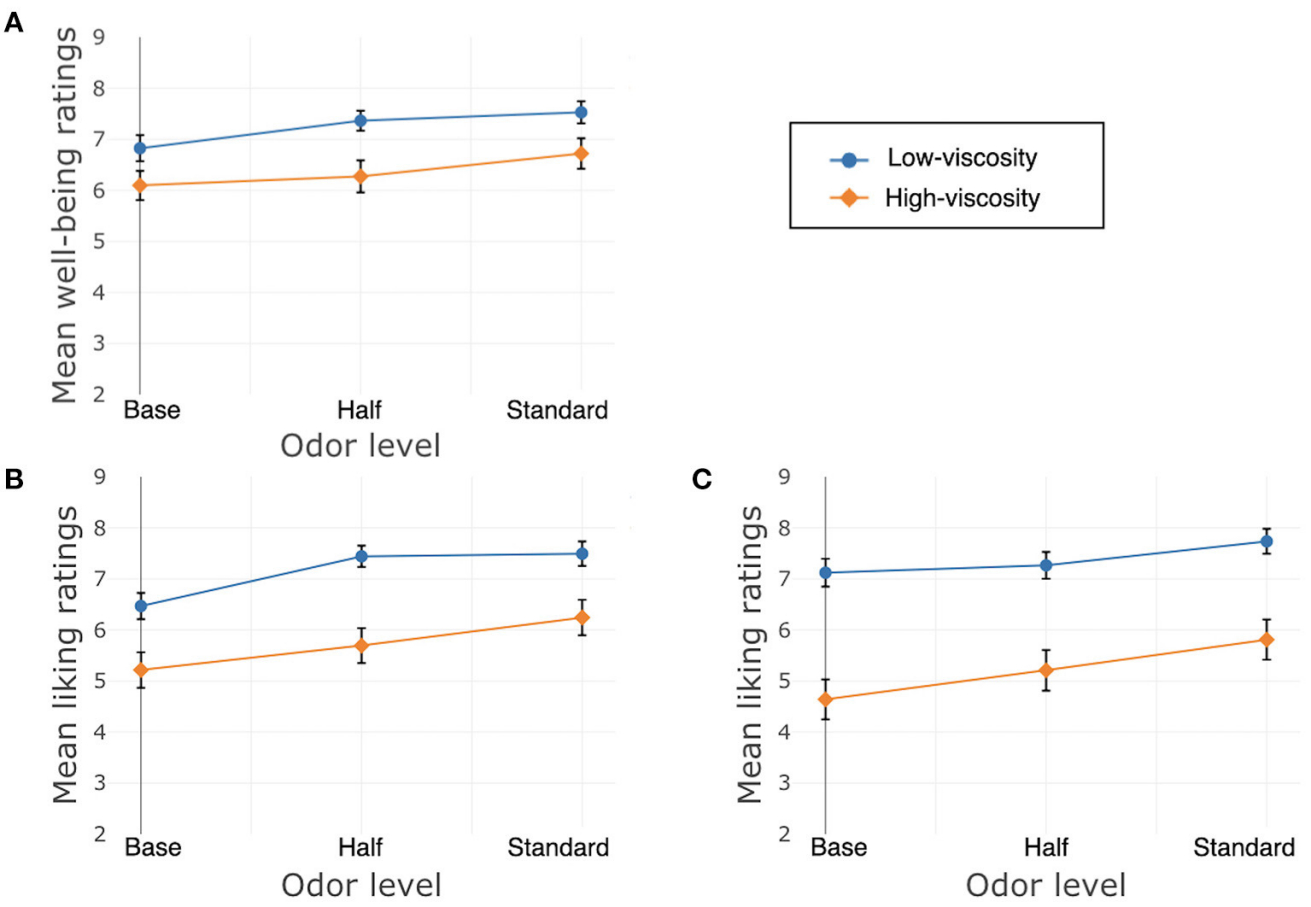
Study 2, France/Face application, **(A)** Well-being/Does me good, **(B)** Liking of the product, **(C)** Liking of the texture. Error bars indicate standard error.

Liking of the texture of the cream was significantly affected only by texture, β = −2.48, *SE* = 0.44, χ^2^(1) = 31.55, *p* < 0.001, again with higher liking ratings for the LV cream. There was no significant interaction between texture and odor, χ^2^(2) = 0.87, *p* = 0.65, and no main effect of odor χ^2^(2) = 2.11, *p* = 0.35. See Figure 5 for well-being and liking ratings.

**Figure 6:**
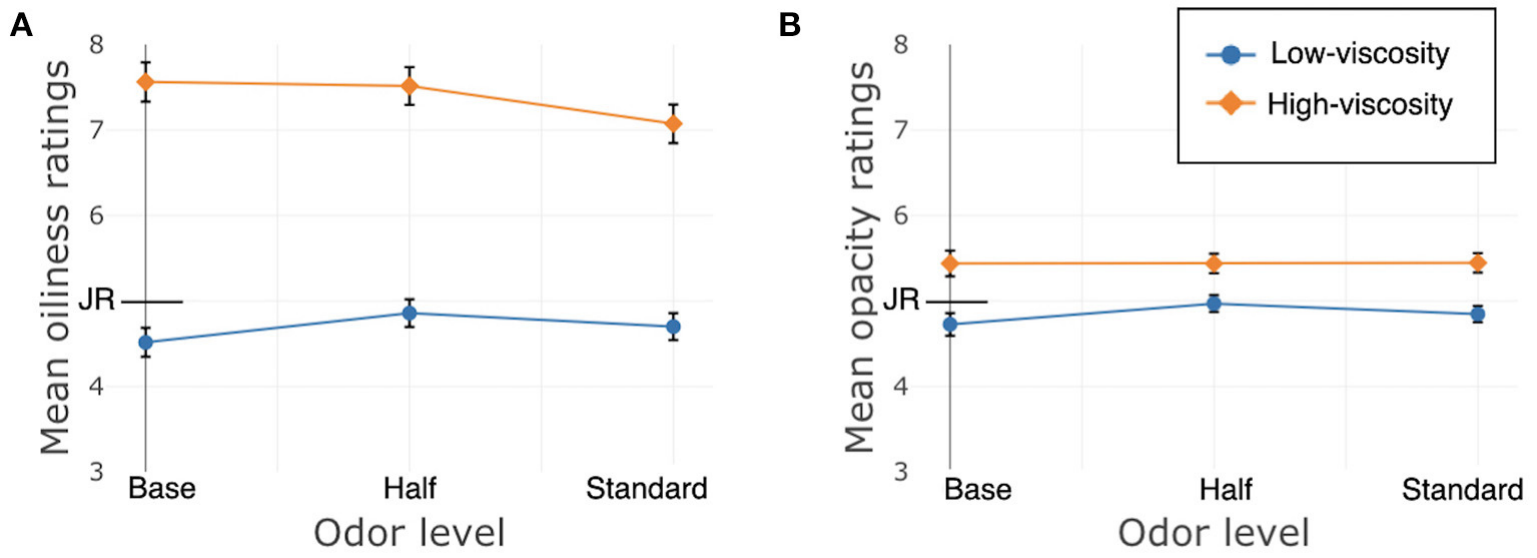
Study 2, France/Face application, JAR ratings. JR, just right. **(A)** Oiliness, **(B)** Opacity. Error bars indicate standard error.

Just About Right (JAR) ratings of oiliness and opacity were affected by texture. Oiliness: β = 3.05, *SE* = 0.23, χ^2^(1) = 173.24, *p* < 0.001; Opacity: β = 0.71, *SE* = 0.14, χ^2^(1) = 25.97, *p* < 0.001. There were no main effects of odor; Oiliness: χ^2^(2) = 2.20, *p* = 0.33; Opacity: χ^2^(2) = 3.04, *p* = 0.22. Nor were there interactions between texture and odor; Oiliness: χ^2^(2) = 4.26, *p* = 0.12; Opacity: χ^2^(2) = 1.52, *p* = 0.47. See Figure 6.

#### Discussion

Results from Study 2 showed higher ratings for “does me some good” after trying the creams with added odor and after trying the LV cream.

As in Study 1, overall liking was affected by both texture and odor. The effect of odor was the same, with participants liking the creams with added odor more than the creams without. However, in contrast to Study 1, participants testing the creams on their faces showed a strong preference for the LV cream. This extended to their liking ratings for texture; only texture influenced these ratings. Participants strongly preferred the LV cream, and the presence of odor did not change their preference.

Participants' JAR ratings were both affected by texture, with the HV cream generally being rated toward too oily and opaque, and the LV cream's ratings around the center of the scale. Odor did not affect these ratings.

The present study has so far examined the interaction of the perception of odor and texture in different contexts (hand and face). Because odor and texture preferences may vary with culture, we wanted to explore whether the effects we found in France could also occur in China, a country with a culture very different from that of France.

The studies were set up as similarly as possible to the studies in France, with one exception: for reasons unconnected to the present report, the authors wished to test a low viscosity lotion with a slightly different texture than the one tested in France. This is the main reason that these different studies have been reported as such rather than comparing French and Chinese participants in a single study. However, all reported data on liking, well-being, and JARs are from questions to which participants in both France and China responded and which were translated to be as similar as possible.

## Study 3: Hand Application In A Chinese Sample

### Materials and Method

#### Participants

Participants were 65 women (M_age_= 41, age range = 30–59). All participants lived in Shanghai and spoke Mandarin. Recruitment criteria were the same as in France. They were recruited by and tested at Biofortis (Mérieux Nutrisciences), a sensory testing laboratory which recruits from a list of participants in the community. Also as in France, participants were unaware of Chanel's involvement in the study, they were reimbursed for their time, all research was conducted according to the principles expressed in the Declaration of Helsinki, written informed consent was obtained from every participant, and the safety of the creams was verified by a toxicologist.

#### Materials

##### Creams

As in the first two studies, the materials used in this study were face creams with two different textures (low and high viscosity), each with three levels of odor. The HV cream was the same as that used in France, with the same three levels of odor, 0 (Base), 0.15% (Half of standard added odor), and 0.3% (Standard added odor). As mentioned above, the LV cream differed because one aim of the overall study (not reported here) was evaluation of this particular product by women in China. Rather than the lotion with *G'* of 220 Pa, participants in China were presented with a lotion with a *G'* of 70 Pa. The standard level of odor for this product is 0.1%, so that was used as Standard in the study. Half was therefore 0.05, and 0% was Base. As in the previous experiments, the odor itself (the same proprietary blend) did not vary, only its concentration, and the 0% condition was not neutral.

##### Questionnaires

The questions reported in Study 1: Hand Application in a French Sample were translated to Mandarin for Study 3. The translations were performed by a French/Chinese professional translator and double-checked by the French and Chinese experimenters of the studies.

#### Procedure

The experimental procedure was the same as in France (Study 1).

### Results

Descriptive statistics (means, SD, ranges) for all the ratings are shown in Table 1. Ratings of well-being after trying the product were only affected by texture, β = 0.78, *SE* = 0.39, χ^2^(1) = 3.29, *p* = 0.047. There was no significant main effect of odor, χ^2^(2) = 4.16, *p* = 0.13, or interaction between texture and odor, χ^2^(2) = 1.31, *p* = 0.52.

Liking of the product was significantly affected by texture, β = 1.56, *SE* = 0.28, χ^2^(1) = 30.47, *p* < 0.001, and odor, χ^2^(2) = 35.56, *p* < 0.001. Using a Bonferroni correction, the model showed significant differences between Standard and Base, β = 1.66, *SE* = 0.28, *t*_(325)_ = 5.89, *p* < 0.001, between Half and Base, β = 1.06, *SE* = 0.28, *t*_(325)_ = 3.74, *p* < 0.001, and between Standard and Half, β = 0.51, *SE* = 0.20, *t*_(325)_ = −2.57, *p* = 0.02. There was also an interaction between texture and odor, with Half and Standard differing from Base, β_half_ = −1.07, SE_half_ = 0.40, β_standard_ = −1.26, SE_standard_ = 0.40, χ^2^(2) = 11.52, *p* = 0.003.

**Figure 7:**
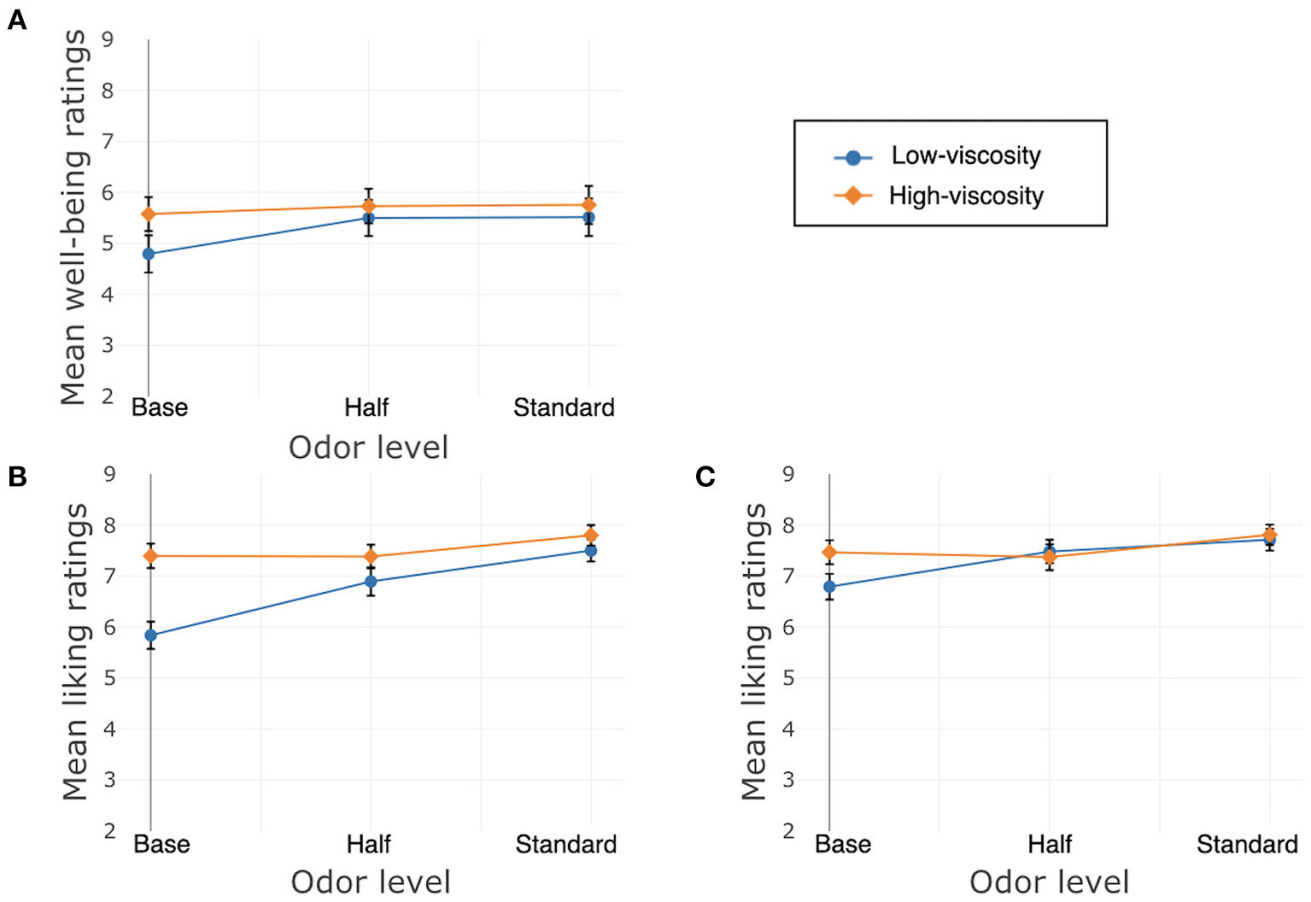
Study 3, China/Hand application, **(A)** well-being, **(B)** liking of the product, **(C)** liking of the texture. Error bars indicate standard error.

Liking of the texture of the cream was significantly affected by texture, β = 0.68, *SE* = 0.27, χ^2^(1) = 6.21, *p* = 0.002, and odor, χ^2^(2) = 12.50, *p* = 0.01. The Bonferroni-corrected model showed differences between Standard and Base, β = 0.92, *SE* = 0.27, *t*_(325)_ = 3.40, *p* = 0.002, and between Half and Base, β = 0.69, *SE* = 0.27, *t*_(325)_ = 2.54, *p* = 0.02, but not between Standard and Half, β = 0.33, *SE* = 0.19, *t*_(325)_ = −1.74, *p* = 0.16. There was no interaction between texture and odor, χ^2^(2) = 4.51, *p* = 0.11. See Figure 7 for well-being and liking ratings.

**Figure 8:**
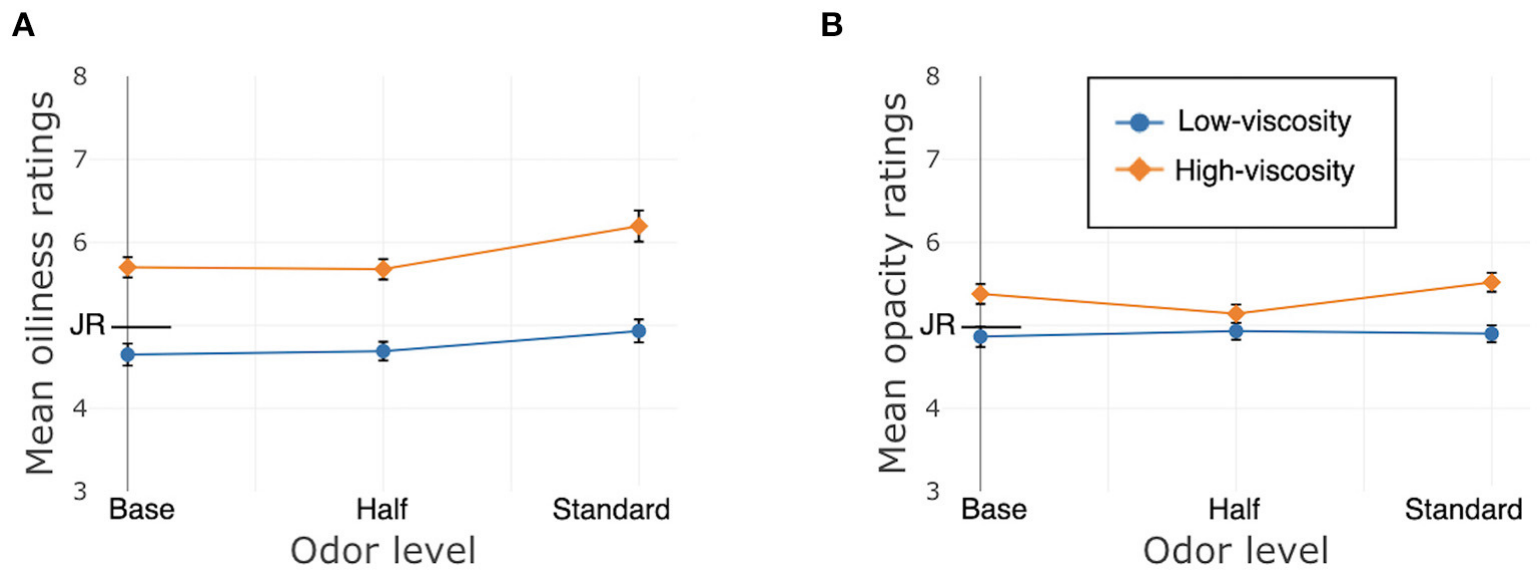
Study 3, China/Hand application, JAR ratings. JR, just right. **(A)** Oiliness, **(B)** Opacity. Error bars indicate standard error.

Just About Right (JAR) ratings of oiliness and opacity were affected by texture; Oiliness: β = 1.05, *SE* = 0.18, χ^2^(1) = 34.45, *p* < 0.001; Opacity: β = 0.51, *SE* = 0.14, χ^2^(1) = 13.22, *p* < 0.001. There were no main effects of odor; Oiliness: χ^2^(2) = 2.93, *p* = 0.23; Opacity: χ^2^(2) = 0.22, *p* = 0.90; nor were there interactions between texture and odor on these ratings; Oiliness: χ^2^(2) = 1.3, *p* = 0.52; Opacity: χ^2^(2) = 4.52, *p* = 0.10. See Figure 8.

### Discussion

Well-being ratings were only affected by texture. There were overall higher ratings for the HV cream. Results on liking of the product and of its texture showed that, though without added odor participants showed a preference for the HV cream, this preference disappeared once odor was added. One important finding was that, even though the LV cream in China had a viscosity that differed from that in France, we still found, as in France, that the addition of odor had a positive effect on ratings of liking. Similar to well-being, JAR ratings were only affected by texture. Average ratings were near the center of the scale, with the HV cream receiving ratings more in the direction of oily/opaque and the LV cream receiving ratings in the other direction.

## Study 4: Face Application In A Chinese Sample

### Materials and Methods

#### Participants

A group of 65 Chinese women completed this task (*M*_age_ = 42, age range = 30–59). Recruitment procedure and criteria and obtaining informed consent were the same as for Study 3. As with Study 2, 32 women were randomly selected to participate in the prosody portion of the study.

#### Materials

##### Creams

The same two creams were used as in Study 3.

##### Questionnaires

The questionnaires were the same as in France (Study 2), translated to Mandarin.

#### Procedure

The experimental procedure was the same as in France (Study 2).

### Results

Descriptive statistics (means, SD, ranges) for all the ratings are shown in Table 1. For the ratings of “does me some good” there was a main effect of odor, χ^2^(2) = 6.56, *p* = 0.04. There was a significant difference between the Standard and Base odor conditions β = 0.43, *SE* = 0.18, *t*_(325)_ = 2.35, *p* = 0.04, the difference between Half and Base approached significance, β = 0.38, *SE* = 0.18, *t*_(325)_ = 2.06, *p* = 0.08, but the difference between Standard and Half was not significant, β = 0.19, *SE* = 0.13, *t*_(325)_ = 1.44, *p* = 0.30. The main effect of texture approached significance, β = 0.33, *SE* = 0.18, χ^2^(1) = 3.27, *p* = 0.07, and there was a significant interaction between texture and odor, with Standard and Half differing from Base, β_Half_ = −0.26, *SE*_Half_ = 0.26, β_Standard_ = −0.74, *SE*_Standard_ = 0.26, χ^2^(2) = 8.45, *p* = 0.02.

Liking of the product was significantly affected by odor, χ^2^(2) = 10.17, *p* = 0.006. Using a Bonferroni correction, the model showed a significant difference between Standard and Base, β = 0.63, *SE* = 0.20, *t*_(325)_ = 3.19, *p* = 0.004, but not between Half and Base, β = 0.30, *SE* = 0.20, *t*_(325)_ = 1.51, *p* = 0.26, or between Standard and Half, β = 0.01, *SE* = 0.14, *t*_(325)_ = −0.08, *p* = 0.93. There was also an interaction between texture and odor, with Standard and Half differing from Base, β_Half_ = −0.06, *SE*_Half_ = 0.28, β_Standard_ = −0.70, *SE*_Standard_ = 0.28, χ^2^(2) = 7.76, *p* = 0.02. There was no main effect of texture, β = 0.14, *SE* = 0.20, χ^2^(1) = 0.51, *p* = 0.48.

**Figure 9:**
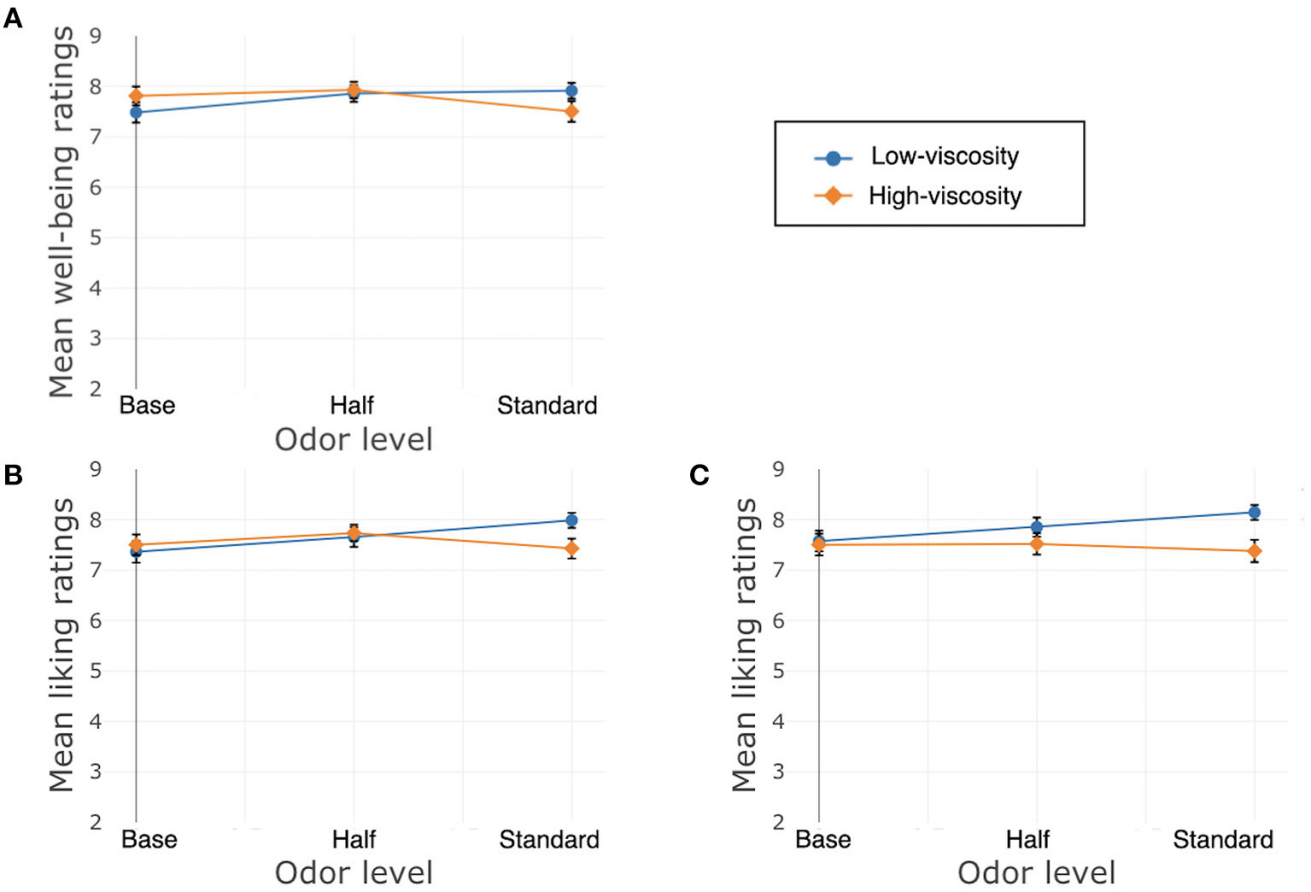
Study 4, China/Hand application, **(A)** Well-being/Does me good. **(B)** Liking of the product. **(C)** Liking of the texture. Error bars indicate standard error.

Liking of the texture of the cream was significantly affected only by odor, χ^2^(2) = 6.09, *p* = 0.048. Using a Bonferroni correction, the model showed a significant difference between Standard and Base β = 0.57, *SE* = 0.23, *t*_(325)_ = 2.47, *p* = 0.03, but not between Half and Base, β = 0.28, *SE* = 0.23, *t*_(325)_ = 1.20, *p* = 0.46, or between Standard and Half odor, β = 0.08, *SE* = 0.16, *t*_(325)_ = −0.46, *p* = 0.64. There was no main effect of texture, β = −0.07, *SE* = 0.23, χ^2^(1) = 0.10, *p* = 0.76, nor was there a significant interaction between odor and texture, χ^2^(2) = 4.64, *p* = 0.10. See Figure 9 for well-being and liking ratings.

**Figure 10:**
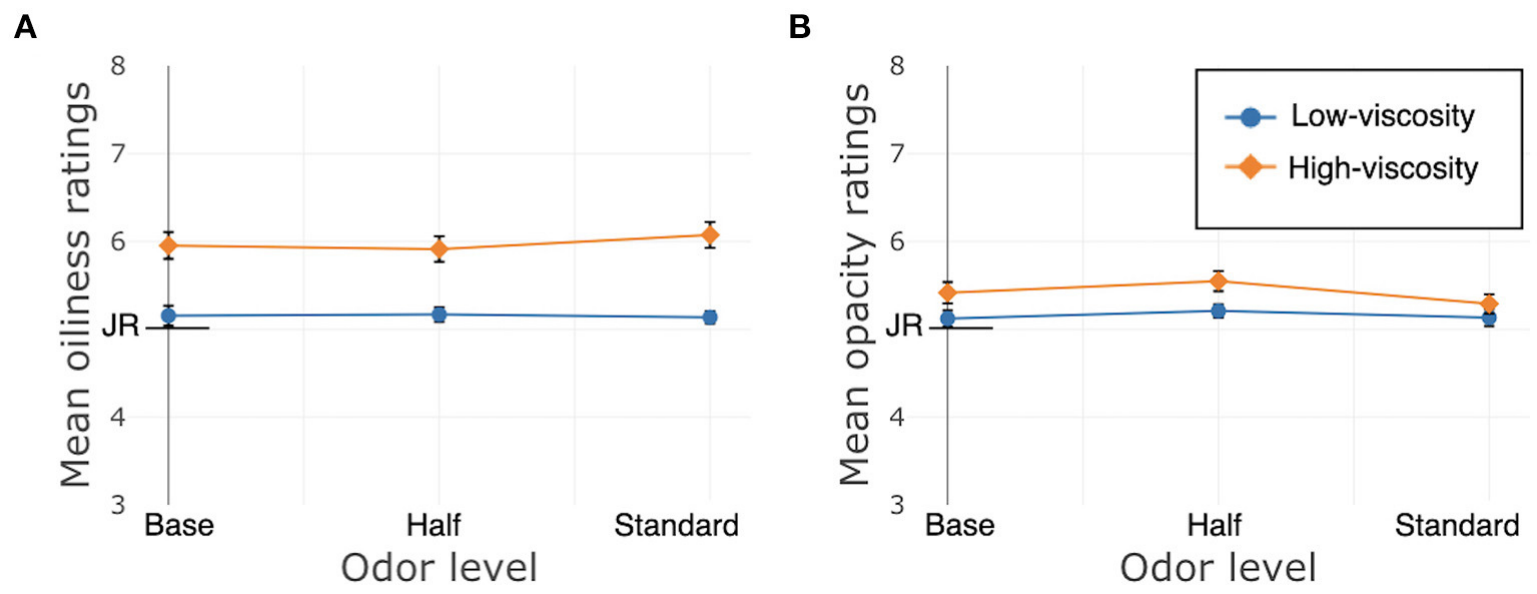
Study 4, China/Hand application, JAR ratings. JR, Just right. **(A)** Oiliness, **(B)** Opacity. Error bars indicate standard error.

Just About Right (JAR) ratings of oiliness and opacity were affected by texture; Oiliness: β = 0.80, *SE* = 0.16, χ^2^(1) = 26.47, *p* < 0.001; Opacity: β = 0.30, *SE* = 0.13, χ^2^(1) = 5.61, *p* = 0.02. There were no main effects of odor; Oiliness: χ^2^(2) = 0.04, *p* = 0.98; Opacity: χ^2^(2) = 0.57, *p* = 0.75; nor were there interactions between texture and odor on these ratings; Oiliness: χ^2^(2) = 0.80, *p* = 0.67; Opacity: χ^2^(2) = 1.18, *p* = 0.56. See Figure 10.

### Discussion

Results show that participants' liking of the product was affected by a combination of the two factors; they preferred the LV cream when there was added odor, but had no preference without odor. Liking of texture showed this same pattern, but only the addition of odor had a significant effect in increasing liking. Similar to liking, ratings of well-being were dependent on the two variables; for the Base odor condition, there was a tendency for higher ratings to be given to the HV cream, but for Standard added odor, higher ratings were given to the LV cream. Here again, even though the LV cream differed in viscosity from that in France, we found effects that were similar to those in France, with the addition of odor increasing liking and well-being ratings.

For the JAR ratings, however, odor had no effect. Only texture determined ratings, with the HV cream rated as oilier and more opaque.

## General Discussion

The results of the present study show that both viscosity and the addition of odor to facial skin care products have strong effects on ratings of well-being, liking, and perception of the products' textures and colors in two differing cultures, France and China. We did not evaluate the effect of texture on ratings of odor, as the scent is likely to have reacted differently with the different components in the two creams with different levels of viscosity. We did, however, find significant effects of odor on ratings of different textures, though there was no evidence that the addition of odor to the cream changed its texture.

### Effects of Added Odor on Well-Being, Liking and JARs

Our first question for the study was how the addition of odor to a cream affected well-being ratings. The addition of odor increased well-being ratings for Studies 1, 2, and 4 (both studies in France and Face Application in China). For the studies in France, this effect appeared straightforward, with the Standard level of odor always resulting in higher well-being ratings than Base. In China, perhaps due to the more extreme difference in texture, there were interactions between odor and texture. In Study 4 (Face Application in China), the addition of odor didn't seem to change ratings for the HV cream, while it did change ratings for the LV cream. In Study 3 (Hand Application in China), well-being was much higher after trying the HV cream relative to the LV cream, which may explain why odor did not have an overall effect in this case.

The second main question was about the effect of added odor on product perception, including liking and JARs. For liking of the product itself, there was a consistent effect of added odor increasing liking ratings. This effect was simple in Study 2 (Face Application in France). There was also an interaction between the factors: for both hand application studies (1 and 3), participants liked the HV more than the LV cream when no odor was added, but the two were liked equivalently with added odor. In Study 4 (Face Application in China), the two textures were equivalently liked without odor, and a preference for LV appeared with odor. Both of these demonstrate a greater effect of added odor on liking for the LV creams relative to the HV cream. The majority of these effects appear when comparing the two added odor conditions to the Base cream. With only one exception (Study 3, liking of the product), there were no significant differences between the Standard and Half added odor levels, suggesting a possible threshold for odor perception, after which increases do not result in greater hedonic experience. This points to one possible direction for future investigations: while the scientific value is evident for studying the emotional effects of varying odors' pleasantness or valence, investigations of emotion that vary odor intensity should not be neglected. Neuroimaging studies have demonstrated differential activation in the amygdala and orbitofrontal regions of the brain depending on whether intensity, valence or both are manipulated (Anderson et al., 2003; Winston et al., 2005), and one recent study has shown a nuanced picture of the effect of odor intensity variation on arousal (Baccarani et al., 2021).

In three of the four studies (1, 3 and 4: Hand Application in France and both studies in China), liking of texture increased with added odor as well. The interactions between texture and odor, that is, the differential effects of odor on liking of the two textures, may be the strongest demonstration of crossmodal perception to arise from this experiment. Textures and odors have often been investigated together in the context of food, but they occur together in cosmetic creams as well. Frequent use of these creams may result in an association between texture and odor by our brains—through the fact that they occur together in space and time, or that they are both pleasant (see Spence, 2011 for a review). Previous studies have used dependent variables such as attention (Spence et al., 2001) or associations between particular odors and colors or shapes, (Deroy et al., 2013; Hanson-Vaux et al., 2013) to investigate crossmodal correspondences. In this case, to investigate the result of texture-odor associations while focusing on the hedonic experience resulting from use of cosmetic creams, we found evidence of crossmodal perception using liking and well-being measures, and, it can even be argued, JAR ratings in one study (1, Hand Application in France).

Both JAR ratings concerned the texture and appearance of the cream. In all of the studies, we found the expected results of the HV cream being rated as oilier and more opaque than the LV cream (ratings which reflected its physical properties). In Study 1 (Hand Application in France), however, there was also an effect of odor: creams with added odor were rated as oilier and more opaque. A possible explanation is that the addition of odor led to participants perceiving the creams as being more luxurious or moisturizing, and oiliness/opacity are accompanying characteristics of luxury (as in Duncan et al., 2020 when participants rated HV creams as more moisturizing than more watery creams). It could also be attributed to a “halo effect” —the term coined by Thorndike (1920) to describe when one salient aspect influences perception of an entire object. Hence, we suggest that this is another instance of crossmodal association: both added scent and oiliness/opacity were associated with luxury or quality, and therefore associated with each other.

The same effect may not have been found on the face because participants had a preference for the LV cream (or at least less of a preference for the HV cream), meaning the oiliness/opacity were not necessarily associated with positive evaluations on the face. One possible explanation for why this effect was not found in China for the hand application study could be because of the higher difference in texture between the two creams. This should be investigated in future studies.

### Comparisons Among Studies

The third question posed in the introduction concerned the differences in ratings between applications on the face and hand. One general difference consistent across both countries was that the participants gave higher liking and well-being ratings after trying the HV cream relative to the LV cream for hands (modulated by odor in France, Study 1) and higher ratings after trying the LV cream relative to the HV cream for their faces (modulated by the presence of odor in China, Study 4).

Even though participants were explicitly instructed in Studies 1 and 3 that they should keep in mind that they were testing a face cream even though it was being applied to their hands, they still showed different liking patterns than the participants in Studies 2 and 4, respectively. Many of these differences could be attributed to physiological differences between the skin of the face and the skin of the hand; for example, the stratum corneum density and barrier are thinner on the face, and the density of nerve fibers is higher than elsewhere on the body (Farage et al., 2014). Sensitivity is also reported more frequently on the face than the hand (Saint-Martory et al., 2008; Berardesca et al., 2013).

Differences between the results of the hand and face studies may in part be due to methodological differences as well. The participants in the hand study only completed two testing sessions and tried three different creams (with varying odor, consistent texture) in each session, whereas the participants in the face study made six separate trips to the testing site and were perhaps less likely to make their ratings based on comparisons with the other levels of odor. They also spent more time completing questionnaires or participating in the prosody study (not reported here), which may have changed their awareness of their own emotional state to be either heightened or fatigued.

Our final question, whether we find similar effects between two cultures, must be answered cautiously given the differences in creams between the studies in the two countries. We can, however, highlight the similarities between the results in the two countries: Odor has effects on ratings of liking and well-being, and its effects interact with texture in interesting ways in both places. Additionally, participants generally seem to have a stronger preference for LV creams on their faces and HV on their hands.

Overall, the results showing the influence of odor on well-being are easy to explain in the context of previous research showing the hedonic effects of odor. The results of odor on liking may also be due to these effects, with the odor's hedonic valence influencing liking, even across modalities. An explanation for its interaction with texture is less obvious given the paucity of research investigating these two senses together. In the present sequence of studies, added odor increased liking ratings for the LV cream more than it did for the HV cream. There has been extensive research in the field of cosmetology on sensory perception and preferences for different viscosities of creams (e.g., Bekker et al., 2013; Kwak et al., 2015), but it remains to be further explored how added odor impacts these preferences.

### Limitations and Future Directions

A limitation of the present report is the methodological differences among the different studies. The testing length differences between the hand and face study arose for two reasons: first, testing multiple creams on the face in the same day was not feasible; the skin cleansing necessary between two creams would likely irritate the facial skin, negatively impacting all emotional measures. This is not the case for the more robust hand skin. Second, returning to the idea that the hand studies replicate a store experience and the face studies the home experience: in a store, it is common to try multiple creams in one session, whereas at home people only use one at a time. Additionally, the difference in cream distribution (given through a syringe in the hand study and letting participants choose their own amount in the face study, also to reinforce these home/store experiences) may have had unintended effects on ratings. The main difference between the procedures in France and China was the LV cream in China having a lower viscosity than the LV cream in France (for reasons connected to a separate investigation). This is why we strove to be cautious when interpreting these data, keeping the methodological differences in mind. Contrasting results from the two sets of studies, such as the JAR ratings not being affected by odor in China while they were in France, could be due to the viscosity differences in the creams being more pronounced, but we cannot conclude this from the present set-up. Equivalent studies in the two countries could be an avenue for future exploration.

One unavoidable limitation is that the base cream, without added odor, did in fact have an odor due to its chemical composition. This is a necessary evil when testing cosmetics; the ingredients that add odor are essential components of the creams. Without them, the textures of the creams would have been nothing like creams women are used to.

One aspect, which is not necessarily a limitation of the study but should be addressed, is that the participants were informed that the creams were premium, luxury products, and this may have biased their perceptions. This was intentional; we wished to test perception of these products among a group of women who habitually used higher-end cosmetics and hence had specific expectations of luxury products.

This study opens up several avenues of further exploration: for example, future work could shift the balance between laboratory-controlled and ecologically-valid tests. We wanted to explore the effects of odor on well-being in a context like that of a cosmetic store, where participants test face creams on their hands, and in a context like that at home, where participants test them on their faces. However, in the present study, both of these explorations were performed in a laboratory setting in order to be able to control ambient odors, lights, sounds, and other aspects of the experience. It would be interesting to evaluate crossmodal interactions while at the same time pushing this a bit further toward a store context, by adding back some of the background noise that would be present at a cosmetics counter in a department store, and/or by having women try the creams in their homes, as part of their morning routine. It would also be interesting to expand the testing sphere to include participants living in more rural regions. In urban settings, people become accustomed to increased levels of pollution, perhaps changing their olfactory perception and/or skin sensitivity. In addition, consumers in urban settings have and are used to having much more variety of skin care readily available to them; they may be more accustomed to the textures and odors present in luxury skin care products.

One possible direction to further explore and refine the results obtained in this study would be to recruit a new group of participants for more in-depth explorations, including focus groups or user journey mapping exercises. It would also be interesting to document user experiences on other types of scales or using visualization exercises to further explore the emotions accompanying use of the creams. Future studies could also investigate the effect of factors external to the cream, such as packaging design, on ratings.

Other possible directions to further explore include varying the odor itself, examining other senses (by varying the color, for example), or exploring odor and crossmodal perception in men. The present study was restricted because the face creams of the type investigated are much more frequently used by women, but many studies have found differences in odor perception or memory between men and women (Lehrner, 1993; Marchand and Arsenault, 2002; Ferdenzi et al., 2013b), while others have not (Larsson et al., 2000; Bengtsson et al., 2001; Kranz et al., 2019), and this could be an interesting avenue to explore. One of these studies (Larsson et al., 2000) found that personality and semantic memory ability had strong effects on odor identification; it could also be interesting to assess whether crossmodal and emotional responses to odor could be affected by these factors.

### Conclusions

The present investigation, the first to our knowledge to examine olfactory-tactile crossmodal perception and well-being, has shown that both odor and texture have effects on well-being and liking of a product, and they interact with each other. Even with variable methodologies, two different cultures, and testing in two different contexts (face/home and hand/store), we found crossmodal effects on well-being, liking, JARs, or a combination of these across all four studies. This means that the effect itself is robust, opening the way for other studies investigating perception of olfactory and tactile stimuli such as other cosmetics or different categories of objects. We also showed that the effects are likely to be context-dependent, given the strong difference in results for HV versus LV creams and the different levels of the odor's influence in the hand and face studies. Future research can build on this study, taking it in numerous different directions to further inform us about olfactory and tactile perception and their interactions.

## Data Availability Statement

The raw data supporting the conclusions of this article will be made available by the authors, without undue reservation.

## Ethics Statement

Ethical review and approval was not required for the study on human participants in accordance with the local legislation and institutional requirements. The patients/participants provided their written informed consent to participate in this study.

## Author Contributions

SC designed and implemented the studies and contributed to the literature review, the statistical analyses and the writeup. AB wrote the manuscript and contributed to the literature review and the statistical analyses. RA performed the statistical analyses, and M-HB supervised the project. All authors contributed to the article and approved the submitted version.

## Conflict of Interest

RA was employed by the company IT&M Stats. AB was employed by the company Morard Editing. The remaining authors declare that the research was conducted in the absence of any commercial or financial relationships that could be construed as a potential conflict of interest.

## Publisher's Note

All claims expressed in this article are solely those of the authors and do not necessarily represent those of their affiliated organizations, or those of the publisher, the editors and the reviewers. Any product that may be evaluated in this article, or claim that may be made by its manufacturer, is not guaranteed or endorsed by the publisher.
